# Effect of High-Protein Diets on Integrated Myofibrillar Protein Synthesis before Anterior Cruciate Ligament Reconstruction: A Randomized Controlled Pilot Study

**DOI:** 10.3390/nu14030563

**Published:** 2022-01-27

**Authors:** Emily E. Howard, Lee M. Margolis, Maya A. Fussell, Clifford G. Rios, Eric M. Meisterling, Christopher J. Lena, Stefan M. Pasiakos, Nancy R. Rodriguez

**Affiliations:** 1 Department of Nutritional Sciences, University of Connecticut, Storrs, CT 06269, USA; emily.e.howard14.civ@mail.mil (E.E.H.); maya.fussell@uconn.edu (M.A.F.); 2 Military Nutrition Division, United States Army Research Institute of Environmental Medicine, Natick, MA 01760, USA; lee.m.margolis.civ@mail.mil; 3 Oak Ridge Institute for Science and Education, Oak Ridge, TN 37830, USA; 4 Orthopedic Associates of Hartford Surgery Center, Glastonbury, CT 06033, USA; crios@oahctmd.com (C.G.R.); clena@oahctmd.com (C.J.L.); 5 Integrated Anesthesia Associates, Hartford, CT 06106, USA; emmmd@reagan.com; 6 Military Performance Division, United States Army Research Institute of Environmental Medicine, Natick, MA 01760, USA; stefan.m.pasiakos.civ@mail.mil

**Keywords:** muscle protein synthesis, disuse atrophy, musculoskeletal injury, anabolic resistance

## Abstract

Increasing dietary protein intake during periods of muscle disuse may mitigate the resulting decline in muscle protein synthesis (MPS). The purpose of this randomized pilot study was to determine the effect of increased protein intake during periods of disuse before anterior cruciate ligament (ACL) reconstruction on myofibrillar protein synthesis (MyoPS), and proteolytic and myogenic gene expression. Six healthy, young males (30 ± 9 y) were randomized to consume a high-quality, optimal protein diet (OP; 1.9 g·kg^−1^·d^−1^) or adequate protein diet (AP; 1.2 g·kg^−1^·d^−1^) for two weeks before ACL reconstruction. Muscle biopsies collected during surgery were used to measure integrated MyoPS during the intervention (via daily deuterium oxide ingestion) and gene expression at the time of surgery. MyoPS tended to be higher, with a large effect size in OP compared to AP (0.71 ± 0.1 and 0.54 ± 0.1%·d^−1^; *p* = 0.076; *g* = 1.56). Markers of proteolysis and myogenesis were not different between groups (*p* > 0.05); however, participants with greater MyoPS exhibited lower levels of MuRF1 gene expression compared to those with lower MyoPS (r = −0.82, *p* = 0.047). The data from this pilot study reveal a potential stimulatory effect of increased daily protein intake on MyoPS during injury-mediated disuse conditions that warrants further investigation.

## 1. Introduction

Muscle atrophy and weakness are well-recognized clinical outcomes of anterior cruciate ligament (ACL) injury and reconstructive surgery that can persist postoperatively despite aggressive physical rehabilitation efforts [1,2]. Muscle atrophy under these conditions results, in part, from knee joint trauma and associated deficits in neuromuscular signaling. Injury-related effusion, inflammation, pain, and damage to articular sensory receptors in the knee joint alter neural signaling from the injured area that restricts activation of surrounding muscle [3,4]. This protective response, combined with periods of limb immobilization and declines in habitual physical activity, induces rapid muscle disuse atrophy due to unloading and reduced neural activation of muscle [5,6]. Developing interventions that mitigate disuse atrophy following ACL injury and subsequent reconstruction may accelerate and optimize recovery.

Disuse atrophy is driven by a persistent negative net muscle protein balance (muscle protein synthesis [MPS] < muscle protein breakdown [MPB]) that results, in part, from decreased postprandial MPS [7]. This “anabolic resistance” to protein ingestion is robust [8,9], and contributes to a rapid suppression of integrated MPS (i.e., measure incorporating fed and fasted states) during periods of disuse [10]. Dietary protein quality (i.e., essential amino acid [EAA] content, digestion and absorption kinetics) and quantity modulate postprandial MPS [11], suggesting protein-based interventions may be used to restore integrated MPS under disuse conditions. Increasing leucine content of the diet enhances integrated MPS in older populations that experience anabolic resistance due to aging [12,13,14]. Manipulating protein intake also modulates MPS in some [15] but not all experimental models of disuse (i.e., limb immobilization and bed rest) [16,17,18]. No study to our knowledge has examined the capacity of protein-based interventions to chronically stimulate MPS during periods of disuse associated with musculoskeletal injury.

Increasing protein intake during periods of disuse before ACL reconstruction may restore anabolism and attenuate muscle loss to better prepare a patient for the metabolic demand for protein during surgery and early postoperative recovery (i.e., maintain ‘reserve’ of amino acids) [19,20]. Protein-based interventions before surgery may also enhance muscle regenerative capacity, as pre-operative EAA supplementation increased satellite cell abundance in total knee arthroplasty patients [21]. The objective of this study was to determine the effect of a high-quality, optimal protein diet (OP; 2.0 g·kg^−1^·d^−1^) compared to adequate protein diet (AP; 1.0 g·kg^−1^·d^−1^) for two weeks before ACL reconstruction on integrated myofibrillar protein synthesis (MyoPS) and proteolytic gene expression. Myogenic regulatory factor gene expression on the day of surgery was a secondary outcome. We hypothesized that OP compared to AP diets would enhance integrated MyoPS during the two-week intervention period, and decrease proteolytic gene expression and enhance myogenic regulatory factor gene expression at the time of surgery.

## 2. Materials and Methods

### 2.1. Participants

Six men aged 19–39 scheduled to undergo their first ACL reconstruction were recruited to participate. Two participating surgeons identified eligible patients that were subsequently screened by study personnel for all eligibility requirements. Exclusion criteria included body mass indexes (BMI) greater than 30, metabolic or cardiovascular abnormalities, food allergies, and gastrointestinal disorders (e.g., lactose intolerance). Individuals that reported using nutritional or herbal supplements, anabolic steroids, and tobacco products were excluded from participation. The study purpose, the experimental protocol, and potential risks were explained to participants before they gave written informed consent to participate. This study was approved by the Institutional Review Boards at Hartford Hospital (Hartford, CT, USA) and the University of Connecticut (Storrs, CT, USA), and was registered with ClinicalTrials.gov as NCT03492021.

### 2.2. Experimental Design

This study was a two-arm parallel-trial design with subjects randomly assigned (1:1 randomization) to AP or OP groups prescribed 1.0 or 2.0 g protein·kg^−1^·d^−1^, respectively, for two weeks before surgery. Daily deuterium oxide (D_2_O) ingestion followed by vastus lateralis muscle biopsies and blood draws during surgery were used to measure integrated MyoPS rates. Muscle biopsies collected during surgery were also used to assess skeletal muscle myogenic and proteolytic gene expression.

### 2.3. Dietary Intake

Participants consumed a eucaloric diet prescribing approximately 1.0 or 2.0 g protein·kg^−1^·d^−1^ (AP or OP), 30% of energy intake from fat, and the remaining calories from carbohydrates beginning two weeks before surgery. Individual energy requirements were established relative to estimated resting energy expenditure (Harris–Benedict equation), level of physical activity, and estimated energy intake reported at baseline. Food intake was assessed at baseline to characterize typical consumption of each macronutrient (i.e., amount and sources). One-week cycle menus were individualized for each participant based on their energy requirements and routine food consumption. Five servings (3 oz each) of beef were provided to participants in the OP group each week as a high-quality protein source. OP and AP participants consumed one serving of a beef-based protein supplement (IsoPrime Beef^TM^) or an isocaloric serving of Powerade^®^ twice a week, respectively. Menus were designed to distribute dietary protein evenly throughout the day (breakfast, lunch, dinner), with OP consuming additional protein as between-meal snacks. A study dietician remained in close contact with participants as they began their individualized study diets and throughout the intervention period. A 7-day food record was collected before surgery to evaluate dietary intake. Participants were provided detailed instructions for filling out the food record, and any discrepancies between prescribed diets and reported intake were reviewed with participants. Food records were analyzed using Nutritionist Pro^TM^ software (Axxya Systems, Woodinville, WA, USA) to estimate total energy, protein, and amino acid content of the diet.

### 2.4. Deuterium Oxide Labeling

D_2_O labeling of the newly synthesized myofibrillar protein fraction was achieved using daily oral consumption of 70% D_2_O. Participants ingested three 50 mL aliquots of 70% D_2_O (150 mL/day) on days 0–7, and two 50 mL aliquots (100 mL/day) on days 8–14 before surgery. All 50 mL aliquots were separated by at least 3 h. This method of D_2_O ingestion rapidly increases and maintains body water enrichment at 1–2% [16,22,23]. Participants recorded the date and time of each D_2_O dose and returned empty bottles to study staff to monitor compliance.

### 2.5. Tissue Sampling during ACL Reconstruction 

Participants were admitted on the morning of their ACL reconstruction in a fasted state. Participants were initially anesthetized with intravenous propofol and maintained with inhalation anesthetic (sevoflurate) during surgery. Regional anesthesia consisting of an adductor canal and geniculate nerve block (0.25% Marcaine with 1:200,000 epinephrine) was also administered. A pneumatic tourniquet was applied as high as possible on the thigh of the injured limb during sterile preparation and draping. A blood sample was obtained and the tourniquet was subsequently inflated to a pressure of 250 mmHG. Vastus lateralis muscle biopsies were collected within 5 min of tourniquet inflation using Rongeur forceps through the incision made for the ACL reconstruction. Muscle tissue was carefully dissected to remove all visible fat and connective tissue, and was snap frozen in liquid nitrogen. All samples were stored at −80 °C until analysis.

### 2.6. Myofibrillar Protein Synthesis

Muscle samples collected during ACL reconstruction were used to measure integrated MyoPS (D_2_O labeling of alanine in myofibrillar protein fraction) during the two weeks before surgery. The myofibrillar protein fraction was isolated from ~50 mg of muscle using methods described previously by Damas et al. [24]. In brief, amino acids from isolated myofibrillar proteins were released by adding 1 mL HCl (1 м) and 1 mL of Dowex resin (50WX8–200 resin; Sigma-Aldrich, Saint Louis, MI, USA) with HCl (0.5 м). Samples were heated at 100° C for 72 h with vortex mixing every 24 h, and subsequently eluted from the resin (2 м NH_4_OH) and evaporated to dryness. Metabolic Solutions Inc. (Nashua, NH, USA) analyzed the muscle preparations for the incorporation of deuterated alanine with a gas chromatograph (GC)—pyrolysis—isotope ratio mass spectrometer (IRMS) (Thermo Finnigan (San Jose, CA, USA) Delta V isotope IRMS coupled to a Thermo Trace GC Ultra with a GC pyrolysis interface III) using previously described methods [24].

Plasma samples were analyzed for D_2_O enrichment by cavity ring-down spectroscopy using a Liquid Water Isotope Analyzer with automated injection system (version 2 upgrade, Los Gatos Research, Mountain View, CA, USA) [25]. Plasma proteins were removed by adding approximately 5 mg zinc sulfate monohydrate to 25–50 µL plasma in a microcentrifuge tube. Samples were vortexed and spun at 8000 rpm to precipitate proteins. The plasma protein-free supernatant was injected eight times and the average of the last three measurements was used for analysis. Standard curves were generated before and after samples to calculate D_2_O enrichment as δ^2^H per mil (‰) relative to Vienna Standard Mean Ocean Water (VSMOW). Intra-run precision was less than 2δ ‰ and inter-run precision was less than 3.5δ ‰. The δ^2^H values were converted to atom percent (Atm%) as previously described [26]:

$$\mathrm{Atm}\%=\frac{100\times AR\times (\delta^{2}H\times 0.001+1)}{1+AR\times (\delta^{2}H\times 0.001+1)}$$

where AR is the absolute ratio constant for D_2_O (0.0015576) and δ^2^H is the value in ‰ to be converted into Atm%.

The myofibrillar protein fractional synthesis rate (FSR, %·day^−1^) was determined by measuring the incorporation of D_2_O-labeled alanine into myofibrillar protein. Plasma-derived body water enrichment (multiplied by 3.7 to account for the exchange of D_2_O between body water and alanine, and divided by 11 to correct for the total number of hydrogens in the derivative) was used as the precursor. FSR was calculated using the standard precursor-product equation:

$$\mathrm{FSR}\;(\%\cdot d^{-1})=[({\mathrm{APE}}_{\mathrm{Ala}})]/[({\mathrm{APE}}_{p})\times t]\times 100$$

where atm% excess (APE)_Ala_ is the D_2_O enrichment of protein-bound alanine, APE_p_ is the mean D_2_O enrichment of total body water (in atm% excess), and *t* is the duration of D_2_O labeling in days. APE_Ala_ and APE_p_ were calculated as day of surgery values minus estimates of baseline myofibrillar protein and plasma D_2_O enrichment (0.0135 and 0.0154%, respectively). Estimates of baseline enrichment were derived from levels previously measured by Metabolic Solutions Inc in a population of young men [24].

### 2.7. mRNA Expression

TRIzol reagent (ThermoFisher, Waltham, MA, USA) was used to isolate total RNA from ~20 mg of muscle to determine expression of several genes linked to skeletal muscle myogenesis and proteolysis. Quantity and quality of isolated RNA were assessed using a NanoDrop ND−2000 spectrophotometer (NanoDrop, Wilmington, DE, USA). Equal amounts of total RNA (500 µg) were reverse-transcribed into cDNA (High-Capacity cDNA RT Kit, Applied Biosystems, Foster City, CA, USA) using a T100^TM^ Thermal Cycler (Bio-Rad, Hercules, CA, USA). mRNA expression of paired box 7 (Pax7), MyoD, Myogenin, myogenic factor 5 (Myf5), myogenic factor 6 (Myf6), muscle atrophy F-box (MAFbx), and muscle RING finger−1 (MuRF1) were determined using commercially available TaqMan^®^ probes (Applied Biosystems). Samples were run in 10 µL reactions in duplicate using TaqMan fast advanced master mix with a Step One Plus Real-Time PCR system (Applied Biosystems). Data were normalized to the geometric mean of β-actin and β2 microglobulin, and expressed for all participants as a fold change relative to AP values using the ΔΔC_T_ method [27]. Sample size for Myf5 was five participants (average CT threshold of 32.8 ± 1.4) since data were not generated for one person in the AP group whose sample failed to cross the CT threshold prior to 40 cycles.

### 2.8. Statistical Analysis

Differences between AP and OP for baseline characteristics, dietary intake, integrated MyoPS, and myogenic and proteolytic gene expression were analyzed using unpaired *t* tests. The association between MyoPS and gene expression was examined using Pearson’s correlation coefficient. Gene data for correlations were log_2_ transformed, since negative fold change means are on a scale of 0–1 while positive data are >1, resulting in uneven scales. Normality was assessed using Shapiro–Wilk tests for dependent variables. Given the small number of participants, effect size was determined as biased corrected Hedge’s *g* with thresholds of 0.2 (small), 0.5 (moderate), and 0.8 (large). All data are presented as the means ± SD. The α level of significance for all statistical tests was two-tailed and set at *p* < 0.05. We also reported values with *p* < 0.1 as trending toward significance. Data were analyzed using IBM SPSS Statistics for Windows version 26 (IBM, Armonk, NY, USA).

## 3. Results

### 3.1. Participants

The 6 male participants were 30 ± 9 years old and had an average BMI of 25.1 ± 2.1 which did not differ between groups (*p* > 0.05; Table 1).

### 3.2. Dietary Intake

Average protein intake derived from dietary records was greater in OP compared to AP (1.9 ± 0.2 g·kg^−1^·d^−1^ and 1.2 g·kg^−1^·d^−1^; *p* < 0.05; Table 2). Total branched chain amino acids, leucine, and valine were also greater in OP compared to AP (*p* < 0.05).

### 3.3. Myofibrillar Protein Synthesis

**Figure 1:**
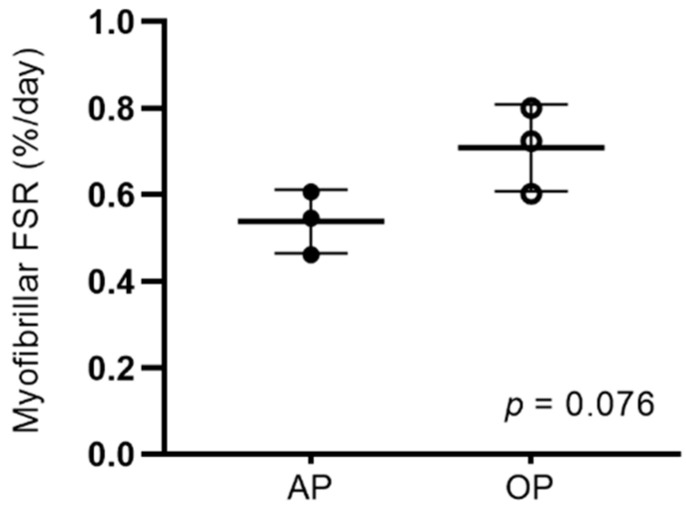
Myofibrillar FSR (%·d^−1^) over a two week period before ACL reconstruction with AP (1.2 g·kg^−1^·d^−1^) and OP (1.9 g·kg^−1^·d^−1^). Differences between AP (*n* = 3) and OP (*n* = 3) were examined using unpaired *t* tests. Values are the mean ± SD. AP, adequate protein; FSR, fractional synthesis rate; OP, optimal protein.

D_2_O enrichment of total body water was not different in OP compared to AP participants on the day of surgery (1.3 ± 0.4% and 1.3 ± 0.4%; *p* = 0.90). MyoPS over the two-week intervention period tended to be greater in OP compared to AP participants (*p* = 0.076; Figure 1). There was a large effect size (*g* = 1.56) for between-group differences in MyoPS.

### 3.4. mRNA Expression

**Figure 2:**
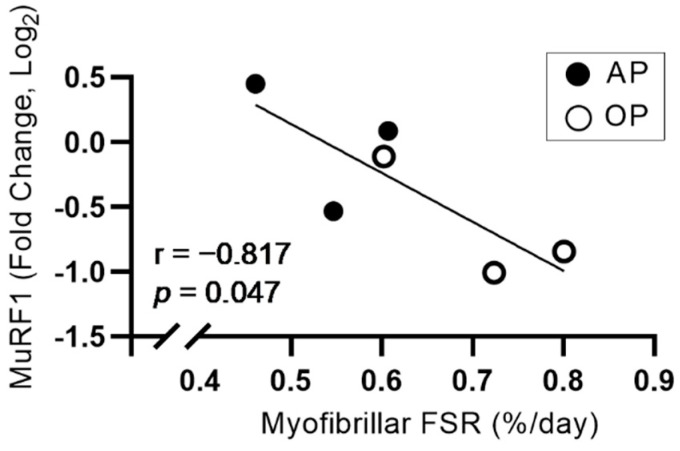
Relationship between myofibrillar FSR (%·d^−1^) and MuRF1 gene expression in AP and OP. Associations were examined using Pearson’s correlation. AP, adequate protein; FSR, fractional synthesis rate; OP, optimal protein.

Myogenic (MyoD, myogenin, Pax7, Myf5, and Myf6) and proteolytic (MAFbx, MuRF1) gene expression was not different between groups (Table 3; *p* > 0.05). There was a large effect size (1.05) for between-group differences in MuRF1 gene expression. MuRF1 gene expression was negatively associated with MyoPS (r = −0.817, *p* = 0.047; Figure 2), and MyoPS was not associated with any other markers of myogenesis or proteolysis (*p* > 0.05; data not shown).

## 4. Discussion

This pilot study showed that patients consuming a high-quality, OP diet (1.9 g protein·kg^−1^·d^−1^) for two weeks before ACL reconstruction tended to have greater MyoPS over the intervention period compared to those consuming an AP diet (1.2 g protein·kg^−1^·d^−1^). While myogenic and proteolytic gene expression were the same in both groups on the day of surgery, MuRF1 expression was inversely associated with MyoPS. These findings suggest a potential stimulatory effect of increased protein intake on MyoPS during injury-mediated disuse conditions.

The large effect size and tendency for increased MyoPS in OP compared to AP is consistent with the stimulatory effect of protein and free amino acids reported in healthy individuals under normal conditions. Oikawa et al. [28] showed that consuming a potato protein supplement twice daily to elevate protein intake from 0.8 to 1.6 g protein·kg^−1^·d^−1^ in healthy young women increased integrated MyoPS by 0.14 ± 0.09%·d^−1^. A comparable difference in protein intake between AP and OP in the current study (0.7 g·kg^−1^·d^−1^) resulted in a similar magnitude of difference in MyoPS (mean difference ± pooled SD; 0.17 ± 0.09%·d^−1^). Contrary to our findings, Kilroe et al. [17] showed that graded protein intakes of 0.15, 0.5, and 1.6 g·kg^−1^·d^−1^ had no effect on MyoPS during three days of experimental disuse (i.e., unilateral leg immobilization). This discrepancy is likely due to differences in diet duration (three days vs. two weeks) or general differences between experimental and injury-mediated disuse conditions (i.e., intramuscular inflammation [29]). Mitchell et al. [16] showed that consuming 20 g of supplemental dairy protein daily during 14 days of immobilization had no effect on MyoPS compared to a placebo. The supplemental protein did attenuate disuse-induced declines in intracellular signaling regulating MyoPS (i.e., mechanistic target of rapamycin [mTOR]-mediated anabolic signaling) [30], suggesting a potential protective effect of increased protein intake during disuse similar to the current study. While some disparities exist, results from previous work collectively support our findings suggesting increased protein intake before ACL reconstruction may have a protective stimulatory effect on MyoPS.

Satellite cell abundance is reduced following ACL injury, which may impair muscle regenerative capacity [31]. Muyskens et al. [21] showed that increasing protein intake by consuming 20 g of EAA twice daily for 7 days before total knee arthroplasty increased muscle satellite cell abundance compared to a placebo. In the current study, markers of myogenesis were not different and had only small or moderate effect sizes between AP and OP groups. This discrepancy may be due to differences in outcomes measured, as we reported gene expression regulating satellite cell activity, while Muyskens et al. used histology and immunofluorescence to report the number of satellite cells in images of muscle tissue sections. Future work should consider examining both outcomes to comprehensively characterize the effect of protein-based interventions on satellite cell abundance and upstream regulation. Transcript levels of the proteolytic markers MAFbx and MuRF1 were also similar in AP and OP in the current study. However, there was a large effect size for decreased MuRF1 gene expression in OP compared to AP (*g* = 1.01), and an inverse association between MyoPS and MuRF1 expression (r = −0.817). These findings support the idea that a high-protein diet stimulating MyoPS before surgery may promote a more favorable anabolic environment than a lower protein diet by attenuating MPB. Since these findings did not extend to MAFbx, and changes in proteolytic gene expression do not always translate to changes in protein content [32] or measured rates of MPB [33], a more comprehensive analysis examining the influence of dietary protein on MPB during periods of disuse before ACL reconstruction is needed.

While this pilot study indicates a potential benefit of consuming OP compared to AP before ACL reconstruction, some limitations must be acknowledged when interpreting these findings and their potential implications. Since this is a pilot study, this work was underpowered to observe statistical differences between groups. Sample size calculations for MyoPS (α = 0.05, β = 0.20) using a mean between-group difference of 0.17%·day^−1^ and a common standard deviation of 0.09%·day^−1^ indicate a sample size of five participants per group may be required to observe a statistical difference. Another potential limitation is the use of historical baseline enrichment values for calculating APE, as temporal drifts in values generated from the same GC-pyrolysis-IRMS may result in underestimation of FSR. This may have contributed to lower FSR in the current work than previously reported [17].

## 5. Conclusions

This pilot study showed that MyoPS tended to be greater in patients consuming a high-quality, OP diet (1.9 g protein·kg^−1^·d^−1^) versus an AP diet (1.2 g protein·kg^−1^·d^−1^) for two weeks before ACL reconstruction. While markers of protein breakdown and muscle regeneration were not different between groups at the time of surgery, patients with greater MyoPS exhibited lower levels of proteolytic gene expression. These data may indicate some benefit of increasing protein intake during periods of disuse before ACL reconstruction; however, the sample size in this pilot study was too small to observe statistical differences. Stimulating MyoPS during periods of injury-mediated disuse may protect muscle mass and optimize recovery. Whether these pilot data translate to a preservation of muscle mass and improved recovery is unknown and should be addressed in future work.

## Figures and Tables

**Table 1:** Participant Characteristics ^1^.

	AP	OP	*p* Value
Age (y)	32 ± 11	30 ± 7	0.78
Height (m)	1.8 ± 0.1	1.8 ± 0.1	0.75
Weight (kg)	85.3 ± 8.2	76.2 ± 14.5	0.45
Body mass index	26.2 ± 1.6	23.9 ± 2.2	0.25

^1^ Values are the mean ± SD. Differences between AP (*n* = 3) and OP (*n* = 3) were examined using unpaired *t* tests and were not different between groups. AP, adequate protein; OP, optimal protein.

**Table 2:** Preoperative Dietary Intake ^1^.

	AP	OP	*p* Value
Energy (kcal/d)	2367 ± 125	2853 ± 382	0.10
Carbohydrate (g·kg^−1^·d^−1^)	3.7 ± 0.6	4.3 ± 1.8	0.60
Fat (g·kg^−1^·d^−1^)	1.0 ± 0.l	1.4 ± 0.3	0.06
Protein (g·kg^−1^·d^−1^)	1.2 ± 0.0	1.9 ± 0.2	0.01
Leucine (mg·kg^−1^·d^−1^)	73 ± 10	104 ± 14	0.04
Isoleucine (mg·kg^−1^·d^−1^)	42 ± 7	58 ± 8	0.06
Valine (mg·kg^−1^·d^−1^)	48 ± 7	68 ± 9	0.04
Total BCAAs (mg·kg^−1^·d^−1^)	162 ± 24	230 ± 32	0.04

^1^ Values are the mean ± SD. Differences between AP (*n* = 3) and OP (*n* = 3) were examined using unpaired *t* tests AP, adequate protein; BCAA, branched chain amino acids; OP, optimal protein.

**Table 3:** Myogenic and proteolytic gene expression ^1^.

	AP	OP	*p* Value	Effect Size
Myogenesis				
MyoD	1.00 ± 0.11	1.53 ± 0.94	0.39	0.63
Myogenin	1.03 ± 0.31	1.12 ± 0.23	0.72	0.25
Pax7	1.20 ± 0.92	0.79 ± 0.16	0.48	0.51
Myf5	1.17 ± 0.85	0.89 ± 0.14	0.60	0.35
Myf6	1.10 ± 0.52	0.96 ± 0.43	0.74	0.24
Proteolysis				
MAFbx	1.10 ± 0.50	0.94 ± 0.24	0.66	0.31
MuRF1	1.04 ± 0.34	0.66 ± 0.23	0.18	1.05

^1^ Values are the mean ± SD. Differences between AP (*n* = 3; *n* = 2 for Myf5) and OP (*n* = 3) were examined using unpaired *t* tests. AP, adequate protein; MAFbx, muscle atrophy F-box; MuRF1, muscle RING finger−1; Myf5, myogenic factor 5; Myf6, myogenic factor 6; OP, optimal protein; Pax7, paired box 7.

## Data Availability

Data are available upon request from corresponding author.
